# A comprehensive evidence-based review on the role of topicals and dressings in the management of skin scarring

**DOI:** 10.1007/s00403-015-1572-0

**Published:** 2015-06-05

**Authors:** G. P. Sidgwick, D. McGeorge, A. Bayat

**Affiliations:** Plastic and Reconstructive Surgery Research, Institute of Inflammation and Repair, Faculty of Medical and Human Sciences, Manchester Institute of Biotechnology, University of Manchester, 131 Princess Street, Manchester, M1 7DN UK; Grosvenor Nuffield Hospital, Wrexham Road, Chester, CH4 7QP England, UK

**Keywords:** Skin scarring, Wound repair, Keloid disease, Hypertrophic scarring, Topical therapy

## Abstract

Wound healing after dermal injury is an imperfect process, inevitably leading to scar formation as the skin re-establishes its integrity. The resulting scars have different characteristics to normal skin, ranging from fine-line asymptomatic scars to problematic scarring including hypertrophic and keloid scars. Scars appear as a different colour to the surrounding skin and can be flat, stretched, depressed or raised, manifesting a range of symptoms including inflammation, erythema, dryness and pruritus, which can result in significant psychosocial impact on patients and their quality of life. In this paper, a comprehensive literature review coupled with an analysis of levels of evidence (LOE) for each published treatment type was conducted. Topical treatments identified include imiquimod, mitomycin C and plant extracts such as onion extract, green tea, *Aloe vera*, vitamin E and D, applied to healing wounds, mature scar tissue or fibrotic scars following revision surgery, or in combination with other more established treatments such as steroid injections and silicone. In total, 39 articles were included, involving 1703 patients. There was limited clinical evidence to support their efficacy; the majority of articles (*n* = 23) were ranked as category 4 LOE, being of limited quality with individual flaws, including low patient numbers, poor randomisation, blinding, and short follow-up periods. As trials were performed in different settings, they were difficult to compare. In conclusion, there is an unmet clinical need for effective solutions to skin scarring, more robust long-term randomised trials and a consensus on a standardised treatment regime to address all aspects of scarring.

## Introduction

The process of wound healing after injury to the skin is complex, with many overlapping mechanisms involved including inflammation, proliferation and tissue remodelling [54]. The inflammation phase takes place in the first 48 h following injury triggering a signalling cascade [50, 77], with neutrophils and macrophages accumulating at the wound site to prevent infection [50, 112]. Fibroblasts begin to synthesise a wide range of extracellular matrix molecules (ECM) during the proliferative phase, repairing the wound and restoring the structure and function of the skin, with new blood vessels formed to restore circulation [35, 104]. The process of remodelling leads to a more formal and cross-linked arrangement of the ECM as the scar matures [100] which can last up to a year [21].

**Fig. 1:**
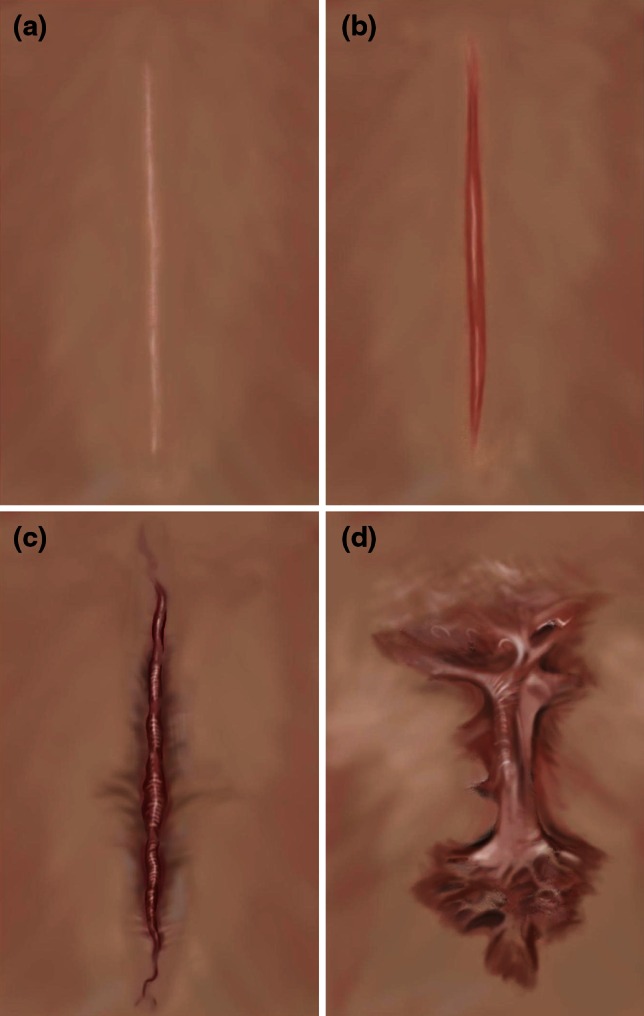
A representation of different types of skin scarring and scar types, as often observed, for example following a mid-sternal incision, post-cardiac surgery. **a** Fine-line scar, **b** hypertrophic scar, **c** intermediate raised dermal scar, **d** keloid scar

Scarring is an imperfect mechanism; an evolutionary compromise made to restore tissue integrity, preventing infection at the expense of appearance. Scars take many forms, depending on size and severity, the type of injury and the anatomical location [7]. Many heal to become fine-line asymptomatic scars, but some lead to abnormal scarring (Fig. 1). There is a spectrum of pathological skin scars ranging from stretched, depressed and/or contracted, to raised dermal scars such as hypertrophic and keloid scars, categorised by over-expression of ECM during the proliferative and remodelling phases of wound healing, which may have a genetic element in certain individuals [16, 19, 99–101]. While sharing characteristics of being raised, keloid scars progress beyond the boundaries of the original wound and do not regress over time unlike hypertrophic scars [21, 70]. Thus, clinical misdiagnosis between the two can complicate findings of clinical trials in management of raised dermal scarring.

Scar scales are used to assess severity from a clinical and patient perspective [45, 95]. These include the Vancouver Scar Scale [108], the Manchester Scar Scale [8], and specialised scales for burns [118] and keloids [83]. All assess symptoms including inflammation, redness (erythema) or colour compared with the surrounding skin, size, scar contour, dryness and itchiness (pruritus). However, no scar scale is perfect; each scale evaluates a different set of criteria using a variable number of criteria, and often relying on the subjective interpretation of individual clinician, making it difficult to compare assessments between studies. The patient-reported impact of scars measure (PRISM) [18] is useful as patients perceive scarring differently to clinicians [41, 46]. This psychological dimension is relevant as skin scarring, which is visible or cannot be hidden by clothing or makeup, can impact on self-esteem and quality of life [15, 17, 111, 114].

Different treatment options are available to aid the scarring process, in particular focusing on severe types of scarring. Some, such as electrical stimulation [20, 49, 84, 90], photodynamic therapy [68, 85] and steroids are only available in a clinical setting. The most cited treatment option for keloids is surgical excision, followed by combination therapy including intralesional steroid injections, most commonly triamcinolone (TAC), with silicone gel or sheeting and/or pressure bandages [13, 102]. However, recurrence rates following keloid revision surgery are high and are difficult to define, varying considerably (40–100 %) depending on the application and adherence to the recommended and prescribed treatment options, the location of the original scar (with keloids excised from the earlobes recurring less frequently than those from the sternum or back) and the follow-up period of the study [2, 16]. Hydration and occlusion, facilitated by silicone gels and sheeting, are thought to potentially influence burn, hypertrophic and keloid scar maturation [1, 26, 92, 97, 117], suppressing the inflammatory response triggered by keratinocytes and the epidermis in response to a compromised stratum corneum [81]. The administration of TAC as a control in combination with other treatments is a common approach to assess efficacy of new treatments; for example, with 5-fluorouracil (5-FU) [3, 38, 52, 115] and verapamil, a calcium channel blocker which stimulates procollagenase synthesis [37, 74].

A number of prescription and over-the-counter topical remedies are available, which claim to alleviate symptoms and improve the appearance of scars and accelerate the rate of wound healing. Topical therapies have their advantages; specifically, increased adherence, the localised delivery of product and the reduced effect of first pass metabolism [107]. Patients often self-medicate independently of clinician care following injury or surgery to treat specific signs and symptoms of concern. Patients only report these concerns and seek diagnosis and treatment, should signs and symptoms worsen, most commonly, in the case of hypertrophic and keloid scarring, leading to a more radical approach being required. There is no comprehensive review of the efficacy of many of these readily available treatments and the quality of research published utilising over-the counter or topical treatments, nor is there a standardised diagnosis and treatment protocol for problematic skin scarring aimed at treating specific signs and symptoms. The aim of this review is to summarise these treatment options and interpret their effectiveness from the published clinical data, in comparison to other approaches, while proposing a more formal approach for treating symptoms of healing wounds, scars and fibrotic scarring such as keloid scarring.

## Methods

A literature search was conducted in Pubmed and Scopus to identify relevant English language literature published in the field of topical treatments for wound healing and keloid and hypertrophic scarring, as alternatives to the commonly utilised silicone and intralesional steroid therapies. Terms included combinations of “wound”, “healing”, “skin”, “scar”, “burn”, “keloid” and “topical”, and were further refined to include the names of individual therapies identified during the search. Key review papers in the field were also consulted. Identified articles were archived in Endnote XI (Thompson Reuters, USA), and duplicates removed. The titles and abstracts of all identified literature were assessed to determine their relevance to the objectives of the review. The reference lists of identified articles were then searched in order to identify further publications of interest. The search focused on clinical trials published since 2000 relating to the effect of skin topicals on healing wounds following surgery, new or mature scars and fibrotic scarring, with an outcome measure of improving the resulting scar quality or reducing the rate of keloid recurrence following revision surgery. Studies in animal or cell culture models were not considered beyond adding context in relation to methods of action. Once identified as being appropriate for inclusion, individual trials were then assessed using the Oxford Level of Evidence (LOE) protocol, to establish the validity and robustness of the data presented (Table 1) [60–62].

**Table 1:** Ranking of studies and clinical trials by their respective levels of evidence (LOE), as defined by the Oxford Centre for Evidence Based Medicine [60–62]

Level of evidence	Study type
1	Systematic review of RCT
	High-quality RCT
2	Systematic review of cohort studies
	Low-quality RCT
	Cohort studies/non-randomised controlled trial
3	Systematic review of case–control studies
	Case–control studies
4	Case series
	Low-quality case–control studies
	Low-quality cohort studies/non-randomised controlled trial
5	Case reports
	Expert opinions

## Results

A considerable range of trials were evaluated for inclusion in this review, from systematic reviews and randomised controlled trials to case/control studies and pilot studies, focussing on different topical treatment options for wound healing, normal scarring and burns as well as for keloid and hypertrophic scars. In some instances individual therapies were trialled on their own, or in combination with more established therapeutic approaches, such as steroid injections, or following revision surgery. These studies were assessed on a case-by-case basis, and were included if the outcome measure demonstrated the benefit or otherwise of the topical therapy intervention compared with standardised treatment—with respect to the quality and properties of the resulting scar as determined by the appropriate scar scales, and in the case of keloid scarring with respect to recurrence following surgery. All trials identified for inclusion are summarised in detail in Table 2. For each treatment option, listed below, an overview is given followed by its effect on the symptoms of normal scarring, burns and fibrotic scarring.

**Table 2:** Systematic ranking of topical treatments reviewed in this article, ranked according to their Oxford Levels of Evidence (LOE), as defined by the Oxford Centre for Evidence Based Medicine [60–62]

References	Sample size/Country of Origin	Study type	Treatment, assessment and follow-up	Outcome—positive or negative findings?	Level of evidence
Berman et al. [10]	*n* = 20, USA	Double-blind, randomised, placebo controlled clinical trial	5 % imiquimod following excision of melanocytic nevi, visual analogue scale used to assess appearance of post-surgical wounds by clinicians and patients. 8 weeks follow-up	Negative/neutral—worse cosmesis evaluation at 8 weeks compared with base cream	2
Martin-Garcia and Busquets [76]	*n* = 6, Puerto Rico	Open-label trial	5 % imiquimod following earlobe keloid excision. Recurrence defined as regrowth of scar tissue surpassing initial excision wound. 8 weeks treatment, 16 weeks follow-up	Positive—prevented recurrence following excision, however, one keloid reoccurred and one responded better to steroids	4
Stashower [105]	*n* = 4, USA	Case study	5 % imiquimod following earlobe keloid shave excision. Recurrence and symptoms assessed visually by lead clinician and patient questionnaire (scar scale utilised not defined). 6 weeks treatment, 12 months follow-up	Positive—no recurrence after 12 months in all 8 keloid lesions	4
Berman and Kaufman [12]	*n* = 12, USA	Case study	5 % imiquimod following earlobe keloid excision, symptoms assessed by clinician (scar scale utilised not defined) 2 months treatment, 24 weeks follow-up	Positive—no recurrence after 24 weeks in patients who completed full follow-up (2 patients lost)	4
Chuangsuwanich and Gunjittisomram [33]	*n* = 45, Thailand	Case study	5 % imiquimod following keloid excision from various sites, recurrence assessed by clinician. 8 weeks treatment, 24 weeks follow-up	Positive—greatest rate of recurrence on chest wall, but earlobe recurrence was least	4
Cacao et al. [23]	*n* = 9, Brazil	Case study	5 % imiquimod following trunk keloid excision. Symptoms assessed by clinician (scar scale utilised not defined) 8 weeks treatment, 20 weeks follow-up	Negative/neutral—all but one keloid excised from the chest/trunk recurred	4
Berman et al. [11]	*n* = 20, USA	Randomised case–control study	5 % imiquimod following keloid shave excision. Symptoms assessed by clinician (scar scale utilised not defined) 6 weeks treatment, 6 months follow-up	Positive—reduction in keloid recurrence vs base cream control, however, not enough patients to determine statistical significance	4
Prado et al. [93]	*n* = 15, Chile	Case–control study	5 % imiquimod following breast surgery, assessed via the Manchester Scar Scale. Treatment duration varied, 24 weeks follow-up	Positive—improvement in treated scar compared with untreated	4
Malhorta et al. [73]	*n* = 2, India	Case study	5 % imiquimod following keloid excision. Symptoms assessed by clinician (scar scale utilised not defined). 8 weeks treatment and 12 weeks follow-up	Negative/neutral—keloids recurred within 12 weeks of stopping imiquimod treatment	5
Patel and Skinner [89]	*n* = 60, USA	Case study	5 % imiquimod following earlobe keloid excision. Symptoms assessed by clinician (scar scale utilised not defined). 8 weeks treatment 24 weeks follow-up	Positive—only one recurrence after 24 weeks	4
Stewart and Kim [106]	*n* = 10, USA	Retrospective case study	Topical mitomycin C following head and neck keloid revision surgery. Recurrence and symptoms assessed by clinician (scar scale utilised not defined). Treatment varied, follow-up 7–14 months	Positive—only one keloid reoccurred	4
Bailey et al. [6]	*n* = 10, UK	Case study	Topical mitomycin C following head and neck keloid shave excision. Patient and clinicians rated symptoms on 10-point analogue scale (scar scale utilised not defined). 6 months follow-up	Positive—only one patient dissatisfied with the response	4
Chi et al. [30]	*n* = 12, Korea	Case study	Topical mitomycin C following earlobe keloid shave excision. Recurrence and symptoms assessed by clinician (scar scale utilised not defined). 12 months follow-up	Positive—only one keloid reoccurred	4
Gupta and Narang [53]	*n* = 20, India	Case study and literature review	Topical mitomycin C following earlobe keloid shave excision. Patient and clinicians rated symptoms on linear analogue scale (scar scale utilised not defined). 6–24 months follow-up	Positive—no keloid recurrence during follow-up period	4
Seo and Sung [98]	*n* = 9, Korea	Case study	Topical mitomycin C following keloid excision. Recurrence and symptoms assessed via Vancouver Scar Scale and patient satisfaction also rated. 6 months follow-up	Positive—six out of nine were satisfied, none were disappointed. Intralesional treatment aggravated the scar	4
Sanders et al. [96]	*n* = 15, USA	Case study—patients had multiple keloids and acted as their own untreated control	Topical mitomycin C following keloid excision, followed by intralesional TAC after 1 month. Recurrence and symptoms assessed by clinician (scar scale utilised not defined). 4–28 months follow-up	Negative/neutral—no difference in response with or without mitomycin C	4
Talmi et al. [110]	*n* = 8, Israel	Case study	Topical mitomycin C following keloid excision. Recurrence and symptoms assessed by clinician (scar scale utilised not defined) and patient satisfaction also rated. 2 months follow-up	Negative/neutral—patients satisfied with outcome, however, total disappearance was only seen in 2 patients	4
Draelos et al. [43]	*n* = 44, USA	Randomised controlled single-blinded trial—patient acted as own untreated control	Topical application of proprietary onion extract cream (Mederma^®^) (4 weeks, once daily) following surgery for seborrheic keratoses. Symptoms assessed by clinician using 4-point scale (scar scale utilised not defined). Follow-up 10 weeks	Positive—significant improvement in appearance compared with treated controls	1
Jenwitheesuk et al. [63]	*n* = 60, Thailand	Randomised, double-blinded placebo controlled trial	Topical application of silicone ± onion extract (Cybele scagel) following surgery, twice daily for 12 weeks. Symptoms assessed via Vancouver Scar Scale and patient satisfaction also rated. 12 weeks follow-up	Positive—using Vancouver Scar Scale, pain itch and pigmentation were improved in onion extract-treated group compared with control	1
Ho et al. [56]	*n* = 120, Hong Kong	Randomised controlled trial	Topical application of onion extract (Contratubex^®^) following laser tattoo removal (twice daily, vs no treatment). Symptoms and appearance assessed by clinician (scar scale utilised not defined). 3 months follow-up	Positive—significantly reduced the risk of scarring from 23.5 to 11.5 %	2
Hosunter et al. [59]	*n* = 60, Turkey	Randomised trial, three groups—onion extract, silicone or both treatments.	Onion extract (Contratubex^®^) on existing hypertrophic and keloid scars, four times per day. Symptoms and appearance assessed by clinician (scar scale utilised not defined). Follow-up 6 months	Negative/neutral—ineffective at improving scar height and itch. Best response was in combination with silicone gel	2
Draelos [42]	*n* = 60, USA	Randomised trial	Topical application of onion extract cream (Mederma^®^) following surgery for seborrheic keratoses. Symptoms and appearance assessed by clinician and patient (scar scale utilised not defined). Follow-up 10 weeks	Positive—improvement in scar appearance compared with untreated controls	3
Chuangsuwanich et al. [32]	*n* = 15, Thailand	Randomised blinded split-scar study	Topical application of onion extract (Cybele scagel) following skin graft. Symptoms assessed via Vancouver Scar Scale and patient satisfaction also rated. 12 weeks follow-up	Positive—improvement in scar scale results compared with untreated control	3
Perez et al. [91]	*n* = 30, USA	Randomised, blinded comparative study	0.5 % hydrocortisone, silicone and vitamin E lotion vs onion extract gel. Keloid and hypertrophic scars. Visual analogue scale used to assess appearance of post-surgical wounds by clinicians and patients. 4 months follow-up	Positive—both treatments more effective than placebo	3
Chanprapaph et al. [27]	*n* = 20, Thailand	Randomised split scar study	Onion extract gel (Erasé gel) on caesarean scars. Visual analogue scale used to assess appearance of post-surgical wounds by clinicians and patients. 12 weeks follow-up	Positive—reduction in mean scar height and symptoms compared with untreated side	4
Koc et al. [69]	*n* = 27, Turkey	Open, randomised comparative study	Onion extract (Contratubex^®^) (three times daily for 3 months) vs intralesional TAC on keloid and hypertrophic scars for 3 months. Symptoms and appearance assessed by clinician and patient (scar scale utilised not defined). 2 months follow-up	Positive—both intralesional TAC and Contractubex were effective, combination therapy was best	4
Karagoz et al. [65]	*n* = 45, Turkey	Randomised case–control study	Comparison of silicone gel (Scarfade^®^), silicone gel sheet (Epi-Derm™) and onion extract (Contratubex^®^) (twice daily) on post-burn scars. Symptoms assessed via Vancouver Scar Scale. Follow-up for 6 months	Negative/neutral—silicone products responded better than Contratubex^®^	4
Chung et al. [34]	*n* = 24, USA	Randomised double-blind split scar study	Comparison of topical petroleum ointment with onion extract cream (Mederma^®^) on fresh surgical wounds, three times daily for 8 weeks. Visual analogue scale used to assess appearance of post-surgical wounds by clinicians and patients. 12 weeks follow-up, telephone interview after 11 months	Negative/neutral—no difference on onion extract treatment compared with petroleum ointment	3
Beuth et al. [14]	*n* = 771, Germany	Retrospective multi-centre cohort study	Onion extract (Contratubex^®^) on post-burn scars and hypertrophic/keloid scars. Symptoms assessed via Vancouver Scar Scale. Follow-up varied	Positive—improved response compared with corticosteroid treatment, with less ADRs	2
Willital and Simon [116]	*n* = 1268, Germany	Observational, non-intervention study	Onion extract (Contratubex^®^) on a range of scars. Symptoms and appearance assessed by clinician and patient (scar scale utilised not defined). Follow-up 4–5 months	Positive—well tolerated, and improvement in scar conditions over follow-up period	2
Muangman et al. [79]	*n* = 63, Thailand	Retrospective case study	Topical application of onion extract (Cybele scagel), twice daily, on partial thickness burns. Symptoms assessed via Vancouver Scar Scale. 24 weeks follow-up	Positive—improves pliability, pigmentation, itch and pain over the study period	3
Campanati et al. [24]	*n* = 35, Italy	Open-label, controlled, non-randomised clinical trial	Topical application of allium cepa–allantoin–pentaglycan gel (Kaloidon gel) to established keloid and hypertrophic scars. Symptoms and appearance assessed by clinician and patient (scar scale utilised not defined). 24 weeks follow-up	Positive—reduced erythema compared with untreated scars. Overall appearance improved	4
Ocampo-Candiani et al. [87]	*n* = 61, Mexico	Randomised controlled trial	Onion extract (Contratubex^®^) following caesarean. Assessed using Patient and Observer Scar Assessment Scale (POSAS). follow-up 12 weeks	Negative/neutral—similar improvement in POSAS results over time with treatment and non-treatment, although Contratubex^®^ was preferred	3
Yoon et al. [119]	*n* = 37, Korea	Randomised split-faced clinical trial	1 or 5 % green tea (*Camellia sinensis*) solution, for acne. Twice daily application vs 3 % ethanol control. Visual analogue scale used to assess appearance of acne by clinicians and patients. 8 weeks study	Positive—severity of acne reduced over 8 weeks study	4
Domingo et al. [40]	*n* = 4, USA	Randomised split-faced clinical trial	2.5 % green tea (*Camellia sinensis*) cream for facial erythema and telangiectasia, twice daily. Symptoms and appearance assessed by clinician and patient (scar scale utilised not defined). 6 weeks study	Positive—no reduction in symptoms however, HIF-1a and VEGF expression was reduced	4
Maenthaisong et al. [72]	Review	Systematic review	*Aloe Vera* for burns—4 studies included, 371 patients utilising a range of assessments and follow-up periods	Negative/neutral—average healing time reduced, however, difficult to compare papers and methodology	2
Eshghi et al. [51]	*n* = 49, Iran	Randomised placebo controlled trial	Topical *Aloe vera* ointment for post-hemorrhoidectomy pain. Visual analogue scale used to assess response by clinicians Treatment three times daily for 4 weeks follow-up	Positive—reduced pain upon defecation and improved wound healing	2
Dat et al. [39]	Review	Systematic review—Cochrane review	*Aloe vera* for treating acute and chronic wounds—7 studies included, 347 patients utilising a range of assessments and follow-up periods	Negative/neutral—evidence that healing time is reduced, however, poor-quality trials make firm conclusions difficult	2
Khorasani et al. [67]	*n* = 30, Iran	Case–control study, each patient acting as their own control	*Aloe vera* compared to silver sulfadiazine for partial thickness burns, twice daily application. Assessment based on percentage area of wound healed over 24 days follow-up	Positive—reduction in average healing time and improved rate of re-epithelisation	3
Khoo et al. [66]	*n* = 122, Malaysia	Randomised double-blinded trial	5 % topical vitamin E on fresh surgical scars, twice daily for 6 weeks (starting 2 weeks after surgery). Assessed using Patient and Observer Scar Assessment Scale (POSAS). 16 weeks follow-up	Negative/neutral—no difference between treated and untreated control	2
Zampieri et al. [120]	*n* = 428, Italy	Randomised singe-blinded case–control study	Topical vitamin E before (three times daily, for 15 days) and after (twice daily for 30 days) surgery in children, to prevent scarring. Symptoms assessed via Vancouver Scar Scale. Follow-up 6 months	Positive—no abnormal scarring and improved cosmetic outcome in test group compared to untreated control	2
van der Veer et al. [113]	*n* = 30, Holland	Randomised, double-blinded placebo controlled trial	Topical calcipotriol (vitamin D) following bilateral mammoplasty, twice daily for 3 months. Symptoms and appearance assessed by clinician (scar scale utilised not defined). 12 months follow-up	Negative/neutral—no difference in scar outcome measures	2
Atiyeh et al. [5]	*n* = 60, Lebanon	Various study types—case/control studies	MEBO^®^ on facial wounds. Visual analogue scale used to assess response by clinicians. 6 months follow-up	Positive—improved cosmetic compared with topical antibiotic (fudicin) and untreated controls	4
Atiyeh et al. [4]	*n* = 66, Lebanon	Various study types—case/control studies	MEBO^®^ following split thickness skin grafts, Visual Analogue Scale used to assess response by clinicians. 6–12 months follow-up depending on study	Positive—improved quality of scar compared with controls	4

Articles identified for inclusion in this review are summarised according to year of publication, country and sample size, along with details of the study design, treatment used and condition, follow-up period and outcome measures. While many of the studies assessed in this review rated scar symptoms such as inflammation, erythema size, scar contour, dryness and pruritus, to determine response to treatment, in many cases these did not relate their findings to a published and recognised specific scar scale

### Imiquimod

Imiquimod is an immune response modifier, typically formulated as a 5 % cream, used in a range of dermatological conditions such as warts and other viral-associated conditions [9]. Ten different articles were identified for inclusion, treating 193 patients [10–12, 23, 33, 73, 76, 89, 93, 105]. Of these studies, just one was a randomised double-blind controlled trial [10] ranked as LOE 2, the rest were case studies and case controlled studies ranked as LOE4. The majority of studies looked at applying imiquimod after keloid shave excision, one followed excision of melanocytic nevi [10] and one following breast surgery. The follow-up period ranged from 8 weeks [10] to 12 months, the remainder of studies (*n* = 8) had follow-up between 16 and 24 weeks or 6 months.

Seven studies led to a positive response, whereas three studies were negative. One case–control study, utilising imiquimod following breast augmentation indicated an improved scar appearance compared with control treatments [93]. A number of different case studies ([9] *n* = 12, [76] *n* = 6, [105] *n* = 4) demonstrated a positive response when used following ear keloid excision, preventing recurrence [12, 76], and improving pruritus, pain and cosmetic appearance over time [105]. The randomised case–control study by Berman et al. [11] following keloid shave excision, demonstrated an improvement, but there were not enough patients (*n* = 20) to demonstrate a statistical significance. The case study by Patel and Skinner [89] demonstrated only one recurrence following ear keloid excision in 60 patients. The case study by Chuansuwanich and Gunjittisomram [33] (*n* = 45) concluded that imiquimod was more effective at preventing recurrence of keloids excised from the earlobe than from other areas, which may be linked to skin tension.

However, the prospective randomised controlled trial by Berman et al. [10] of imiquimod following surgical excision of melanocytic nevi (*n* = 20) found no clinical or cosmetic short-term benefit. The case study by Malhorta et al. [73] (*n* = 2) indicated that discontinuation of imiquimod following presternal keloid excision led to recurrence within 4 weeks, although with the limited sample size, it is difficult to draw firm conclusions. The case study by Cacao et al. [23] (*n* = 9) concluded that imiquimod failed to prevent recurrence after 20 weeks following surgical excision of trunk keloids, although this could be related to anatomical location as many of the studies that indicated a positive response were following keloid excision from the earlobe.

### Mitomycin C

Mitomycin C is an anti-tumour antibiotic, which inhibits DNA synthesis and cell proliferation, and is used to prevent recurrence following keloid scar excision. Mitomycin C inhibits keloid fibroblast proliferation in a cell culture model [103], and was shown to improve response and reduce scarring following aerodigestive surgery [94]. While this can be administered intralesionally, a number of preliminary topical trials have been performed, applying mitomycin C for 2–4 min directly following keloid excision. Seven different articles were identified for inclusion, treating 84 patients in total [6, 30, 53, 96, 98, 106, 110]. All of these were small case studies for treating keloids following surgical or shave excision, classified with an LOE of 4. The shortest follow-up period was 2 months, the remainder were all 6 months or more. Five studies led to a positive response, whereas two studies drew neutral or negative conclusions.

A retrospective case study, utilising mitomycin C following excision of head and neck keloids (*n* = 10) indicated that recurrence was prevented in all but one patient [106]. Several case studies following earlobe keloid shave excision (Bailey *n* = 10, Chi *n* = 12 and Gupta *n* = 20) concluded that patients and clinicians were satisfied with the outcome, with scar improvement in almost all cases [6, 30, 53]. A further case series conducted by Seo and Sung [98] (*n* = 9) concluded that topical mitomycin C treatment led to a more favourable response, while wounds treated with intralesional mitomycin C responded worse. However, the case study by Sanders et al. [96] (*n* = 15) showed no difference in keloid recurrence treated with or without mitomycin C in combination with intralesional TAC. In the case study by Talmi et al. [110] (*n* = 8), despite patients appearing satisfied, mitomycin C failed to prevent recurrence.

### Plant extracts

A number of plant extracts, with a basis in traditional medicine, marketed as “natural” alternatives, have been used in a range of wound healing and cosmeceutical formulations. Many are used in combination with other treatment regimes, either formulated in creams or added to dressings, to try and improve the conditions for wound healing.

### Onion extract

Onion extract contains a range of phenolic anti-oxidant and anti-inflammatory compounds and were originally used for treating full and partial thickness burns; however, more recently these have been trialled for the treatment of hypertrophic and keloid scarring, and healing wounds. Onion extract and quercetin have been shown to reduced fibroblast proliferation in a cell culture mode, inducing matrix metalloproteinase-1 expression, suggesting a role in ECM remodelling [31]. For topical treatments for skin scarring containing onion extract, 16 different articles were considered for inclusion, treating 2,703 patients [14, 24, 27, 32, 34, 42, 43, 56, 59, 63, 65, 69, 79, 87, 91, 116]. Of these, however, two papers [14, 116], both ranked as LOE category 2 were large retrospective analysis, with 771 and 1269 patients, respectively, and a third was a retrospective case study [79], ranked as LOE 3 with 63 patients. Of the remaining 13 articles (*n* = 600), two were randomised controlled trials [43, 63] ranked as LOE category 1, two were randomised trials ranked as category 2 [42, 59] and the remainder were smaller, randomised case–control studies of varying sizes, ranked as LOE category 3 and 4. Follow-up period ranged 2–6 months, with most (*n* = 8) being 10–12 weeks.

The most commonly utilised onion extract products are Mederma^®^ [34, 42, 43] and Contractubex^®^ [14, 56, 59, 65, 69, 87, 116], (both Merz Pharmaceuticals, LLC, Greensboro, North Carolina) Mederma^®^ is the US formulation containing 10 % aqueous onion extract and 1 % allantoin, whereas in addition, Contractubex^®^ (the European formulation) contains 50 U heparin per gramme. The remaining studies utilised Cybele^®^ Scagel [32, 63, 79] (Bangkok Botanica, Bangkok, Thailand), Erasé gel [27], (ABCA Pharma Lab Co., Ltd., Nonthaburi, Thailand) and Kaloidon gel [24] (Laboratori Farmacologici Milanesi, Milan, Italy).

Eleven of the studies identified reported positive experiences with onion extract. Both of the large retrospective studies concluded that Contractubex^**®**^ was well tolerated, leading to an improved scar condition as assessed using a range of scar scales and patient opinion over time [14, 116]. The randomised controlled trial by Ho et al. [56] (*n* = 120) using Contractubex^**®**^ following laser-assisted tattoo removal observed reducing scarring compared with untreated control. Two randomised studies by Draelos in 2008 (*n* = 60) and 2012 (*n* = 44), following shave excision of seborrheic keratoses, concluded that Mederma^**®**^ improved the appearance, signs and symptoms of the healed wounds compared with untreated controls [42, 43]; however, both these studies note that funding was provided by the manufacturer Merz Pharmaceuticals. The split-scar analysis by Chanprapaph utilising Erasé gel following caesarean (*n* = 20) indicated improvement in scar height and symptoms in the treated half, but no difference in redness, pliability or overall appearance [27]. The three studies ultilising Cybele^®^ Scagel following excision of presternal hypertrophic scars ([63], *n* = 60), skin grafts ([32], *n* = 15) and partial thickness burns ([79], *n* = 63) all concluded that the treatment led to a better response, with respect to scar appearance and symptoms, than untreated wounds. The open-label non-randomised trial by Campanati et al. [24] (*n* = 35) which used Kaloidon gel on established keloid and hypertrophic scars concluded that erythema was reduced and overall appearance improved compared with the untreated controls. The randomised blinded comparative study by Perez (*n* = 30), comparing onion extract gel with a hydrocortisone, silicone and vitamin E lotion to treat keloids and hypertrophic scars, concluded both were more effective than placebo; improving appearance, lesion induration and pigmentation. However, hydrocortisone was more effective at improving erythema and pigmentation [91].

Two studies concluded that combination therapy led to improved scar response than individual treatments. The open, randomised comparative study by Koc et al. [69] (*n* = 27) determined that the combination of Contractubex^**®**^ and intralesional TAC was more effective at relieving pain and itching in hypertrophic scars and keloids than TAC alone. The comparative study by Hosunter et al. [59] (*n* = 60) using Contractubex^**®**^ and silicone gel sheeting to treat keloid and hypertrophic scars concluded that co-administration led to the best response.

Three studies gave neutral or negative conclusions. The randomised case–control study by Karagoz (*n* = 45) in hypertrophic burn scars concluded that silicone gel sheeting was more effective than Contractubex^**®**^ at improving appearance and condition [65]. The randomised double-blind split scar study by Chung et al. [34] (*n* = 24) following surgery, with Mederma^**®**^ compared with petrolatum emollient found no difference between the two treatments. The randomised controlled trial by Ocampo-Candaini (*n* = 61) following caesarean determined that while patients liked Contractubex^®^, there was no difference in the improvement in POSAS scale scores over time compared with no treatment; however, follow-up was only 12 weeks in this study which may not have been enough time to observe a significant response.

### Green tea

Green tea (*Camellia sinensis*) contains phenolic compounds, known as catechins, with anti-oxidant and anti-inflammatory properties. Popular in traditional medicine and as a beverage, topical application is thought to provide a range of benefits, including a chemoprotective effect against UV radiation [48]. Studies have demonstrated the positive effect of the green tea polyphenol (−)-epigallocatechin-3-gallate (EGCG) in a keloid fibroblast culture model, explaining its potential benefit in vivo. One study showed significant inhibition of mast cell-stimulated type I collagen expression via blocking of the PI-3K/AkT signalling pathways [121], another demonstrated significant suppression of collagen production and proliferation via inhibition of the STAT3-signalling pathway [88]. In a punch biopsy, ex vivo culture model, EGCG was shown to significantly inhibit growth and induce keloid shrinkage [109].

Studies performed in animal models [29, 71] demonstrated that EGCG has a positive effect on wound healing; nevertheless, clinical trials in human subjects in the domain of wound healing and scarring remain to be established. Two trials were identified for inclusion in this study [40, 119] with a total of 41 patients. Both were ranked as LOE category 4. The randomised split-faced trial in acne sufferers by Yoon [119] (*n* = 37) demonstrated that EGCG was well tolerated, and effective at reducing symptoms and inhibiting *P. Acnes* over the 8-week study period. Another split-face pilot study by Domingo in patients with erythema and telangiectasia (*n* = 4) demonstrated that although symptoms did not improve, HIF-1α and VEGF expression were reduced compared to vehicular control [40].

### *Aloe vera*

*A. vera* has been used in traditional medicine for centuries; however, the literature available for its use in wound healing and skin scarring in particular is lacking. Following the literature search, four articles were identified for inclusion in this review, two of which were systematic reviews. One of these, a recent Cochrane systematic review [39] (*A. vera* for treating acute and chronic wounds, identifying 7 studies, involving 347 patients) concluded that clinical evidence for the utility of *A. vera* in treating acute and chronic wounds was mixed, and that firm conclusions of its effectiveness in improving the rate of wound healing or the quality of the scar produced was lacking due to the absence of high-quality trials and the range of different treatment settings and assessment criteria utilised. Another systematic review [72], focussed on the use of *A. vera* for treating burns, identified 4 studies with 371 patients. Although this study had found that average healing time and the re-epithelialisation rate appeared to be reduced, there was no assessment of improvement or otherwise in scar quality. As also concluded by other systematic reviews included in this review, it was difficult to compare the various methodologies and papers to draw firm conclusions as to the effectiveness of *A. vera* in this context.

Of the two remaining studies involving 79 patients, one was ranked as LOE category 2 [51] and one as category 3 (Khorasani 2009 [67]), with both reaching positive conclusions in terms of the rate of initial wound healing observed. The first, a randomised placebo controlled trial utilising *A. vera* cream following hemorrhoidectomy (*n* = 49), concluded that pain was reduced and the rate of wound healing increased initially compared with placebo treatment after 2 weeks, but after 4 weeks, the maximum follow-up period of this study, there was no significant difference between the two groups. No assessment of final scar quality was made, with 4 weeks being too short a time period for any conclusions regarding mature scar tissue to be of relevance [51]. The second, a case–control study of *A. vera* compared with silver sulfadiazine cream for the treatment of second-degree burns (*n* = 30), indicated that the rate of re-epithelisation and healing was greater with *A. vera.* However, no assessment of healed burn scar quality with respect to silver sulfadiazine control was made, and follow-up was only 24 days [67].

### Vitamin E

Vitamins have been used in topical cosmeceuticals and moisturisers for decades, as an aid to improving skin condition, and their anti-oxidant properties are thought to help prevent UV damage in photo-aged skin. The most commonly cited is vitamin E (tocotrienol). One review concluded that evidence for its efficacy in wound healing was lacking [55]. A survey and review of the use of vitamin E to aid skin scarring concluded that although it is often recommended by clinicians, evidence in the literature for its efficacy is lacking [36].

Two studies utilising vitamin E following surgery were identified [66, 120], totalling 550 patients. Both were ranked as LOE category 2. The large prospective randomised single-blinded study by Zampieri (*n* = 428) assessed vitamin E both pre- and post-surgery in children. Utilising the Vancouver Scar Scale, they summarised that treated wounds were of better appearance with less problematic scarring than the placebo group after 6 months [120]; however, this paper was criticised in relation to their definition of keloid scarring, which should not be as prevalent in young children of Italian ethnicity [82]. The double-blind trial by Khoo following surgery (*n* = 122) observed no change in overall scar appearance compared with the placebo after 16 weeks [66].

### Others

Several other studies were identified which looked at the effect of topical treatments on skin scarring as primary outcome. A randomised double-blind placebo controlled trial by van der Veer et al. [113] (ranked LOE level 2) of the use of topical vitamin D (calcipotriol) twice daily for 3 months following bilateral reduction mammoplasty (*n* = 30) concluded that it had no effect in reducing the occurrence of hypertrophic scarring compared with control over the 12-month follow-up period.

Another commercially available product is moist exposure burn ointment (MEBO^®^, Julphar Gulf Pharmaceutical Industries, Ras Al-Khaimah, UAE). While this is most commonly used for burns, two prospective studies utilised MEBO^®^ to improve scar quality [5] and wound healing [4] were identified (*n* = 126). Both were ranked as LOE level 4, each comprising a range of individual case studies and small randomised blinded trials with 6 months follow-up periods. The first (*n* = 60) concluded that cosmetic appearance of scars treated was improved when compared with topical antibiotic (fudicin) and untreated controls. The second (*n* = 66), in split thickness skin grafts or following facial surgery, also observed improved wound healing and scar formation. A new formulation, MEBO SCAReducer^®^, is now also available from the same manufacturer.

## Discussion

The studies identified for inclusion in this review were assessed according to their design and data by the rankings defined by the Oxford Levels of Evidence (Table 2) [60–62]. In summary, 39 articles were identified (excluding systematic reviews and retrospective studies), with 1703 patients. The majority of articles were categorised as Level 4 (*n* = 23), with a relatively small number, less than 25 %, being classified as robust studies (LOE 1 or 2, *n* = 9). In all cases, there was conflicting evidence as to whether the topical intervention was of benefit.

Several large systematic Cochrane Reviews have been published, in the use of silicone gel sheeting for the prevention of hypertrophic and keloid scars ([86]; 15 trials, involving 615 patients), *A. vera* for treating acute and chronic wounds ([39]; 7 trials, 347 patients), topical treatment for facial burns ([57]; 5 RCT, 119 patients) and honey as a topical treatment for wounds ([64], 25 trials with a total of 2987 participants). The main conclusion drawn from these reviews was that while several options are available for treatment of a range of different scar types, many of which are used extensively, there were no large-scale studies with prolonged follow-up periods to draw firm conclusions regarding long-term efficacy [44], a recommendation which is also made by this review. Common problems encountered during the preparation of this review included the limited quality and individual flaws with much of the available literature, in particular low patient numbers, poor randomisation and blinding, the range of different scar assessment methodologies used and outcome measures reported, and short follow-up periods. Results from clinical trials should be looked at cautiously, especially due to the number of positively associated clinical trials identified and the levels of evidence presented, as these may be subject to selection bias where negative results are not published and therefore are not available to review [47, 58].

Despite the volume of research into treatments for skin scarring, there is little evidence to support many over-the-counter treatments and cosmeceuticals available [122]. A recent review concluded many of the advertising claims made by these products cannot be substantiated [78]. This is a common finding for many cosmeceuticals, as there is no requirement to undertake clinical research, making it difficult to critically evaluate the available evidence in comparison to other approaches listed in this review.

It is difficult to randomise a trial based on wound healing, or to compare between studies performed in different patient subsets and hospitals. Many factors such as anatomical location, patient demographics and medical history, surgical operation performed or the age and type of scar, the injury that caused it and the lack of controls, are impossible to standardise between trials [22]. Anatomical location is of particular relevance with revision surgery for keloid scarring; as summarised in the literature above, the greatest rate of success across all treatments was with earlobe keloids, with recurrence of keloids excised from the sternum being most problematic. The follow-up period for such studies should ideally be 12 months or more, to ensure that the observed effect is due to treatment and not the natural process of wound maturation which improves over time; the majority of trials identified for inclusion in this paper had follow-up periods of 6 months or less. A further factor in interpreting the literature is the variability in scar scales utilised and the subjective nature of assessment; while many of the studies assessed in this review rated scar symptoms such as inflammation, erythema, size, scar contour, dryness and pruritus, in many cases these did not relate their findings to a published and recognised specific scar scale. In the literature cited in this review (see Table 2 for references), the Vancouver Scar Scale [108] was one of the commonly cited assessments of scar symptoms [14, 32, 63, 65, 79, 98, 120]. This variability made it particularly challenging to directly compare and contrast the various studies identified. The subjective nature of scar assessment could be limited by having different clinicians independently analyse the same scars in each study. An alternative issue is that many formulations contain multiple ingredients, and their efficacy in combination is difficult to elucidate in the limited trials performed. There are examples in the literature where a combination of different approaches have shown success [25], indicating how difficult the management of keloids and problematic scarring can be.

There is an unmet clinical need for effective treatments for skin scarring, in particular to address inflammation, pruritus, dryness and redness, commonly cited by patients as the factors which affect them most. Furthermore, robust and consistent, large scale, clinical trials are required to determine the effectiveness of these treatments. A number of articles have summarised different treatment options available for wound healing and scarring; however, these focus on diagnosis and management, and do not review the role of topical therapies in detail [2, 28, 75, 80]. These currently recommend topical silicone gel or dressings along with intralesional steroids and pressure therapy for problematic scarring, and potential surgical revision if required. Based on the current levels of evidence presented in this review, it is difficult to recommend the topical treatments identified as alternatives to current practice based on the limited clinical trial data currently available, although some success has been shown when these are utilised as supplementary therapy to standard practice in addressing specific symptoms of concern.

**Fig. 2:**
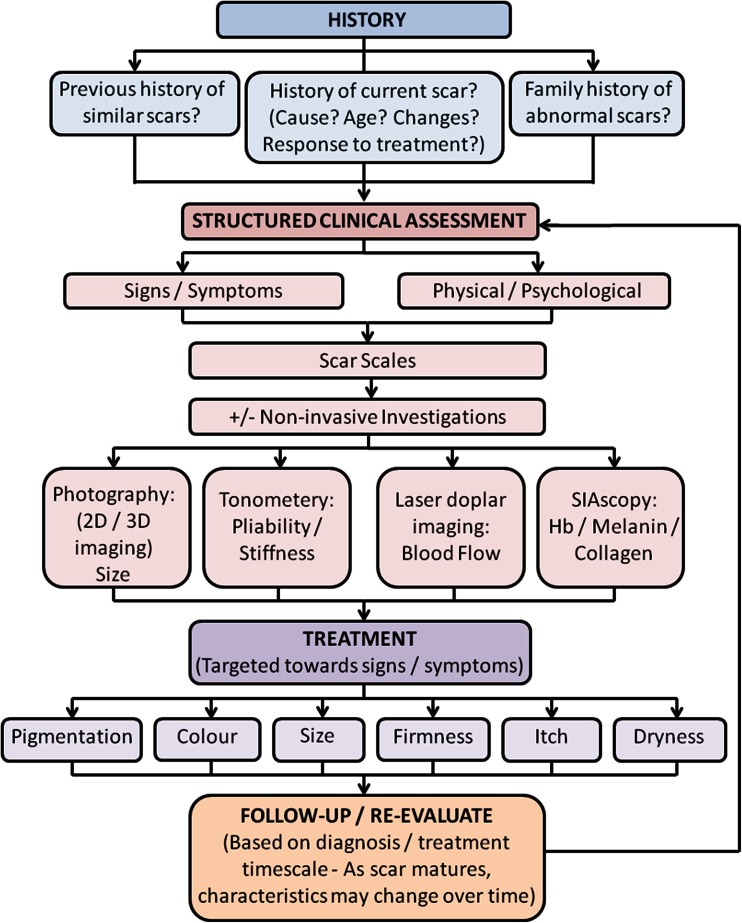
A proposed flowchart indicating the different stages of the scar management timeline, as recommended by the corresponding senior author. A structured clinical assessment is required, taking into consideration patient medical and family history, including current signs and symptoms and utilising a range of quantitative and qualitative measurements in order to enable a targeted treatment, which may evolve over time as signs and symptoms change

**Fig. 3:**
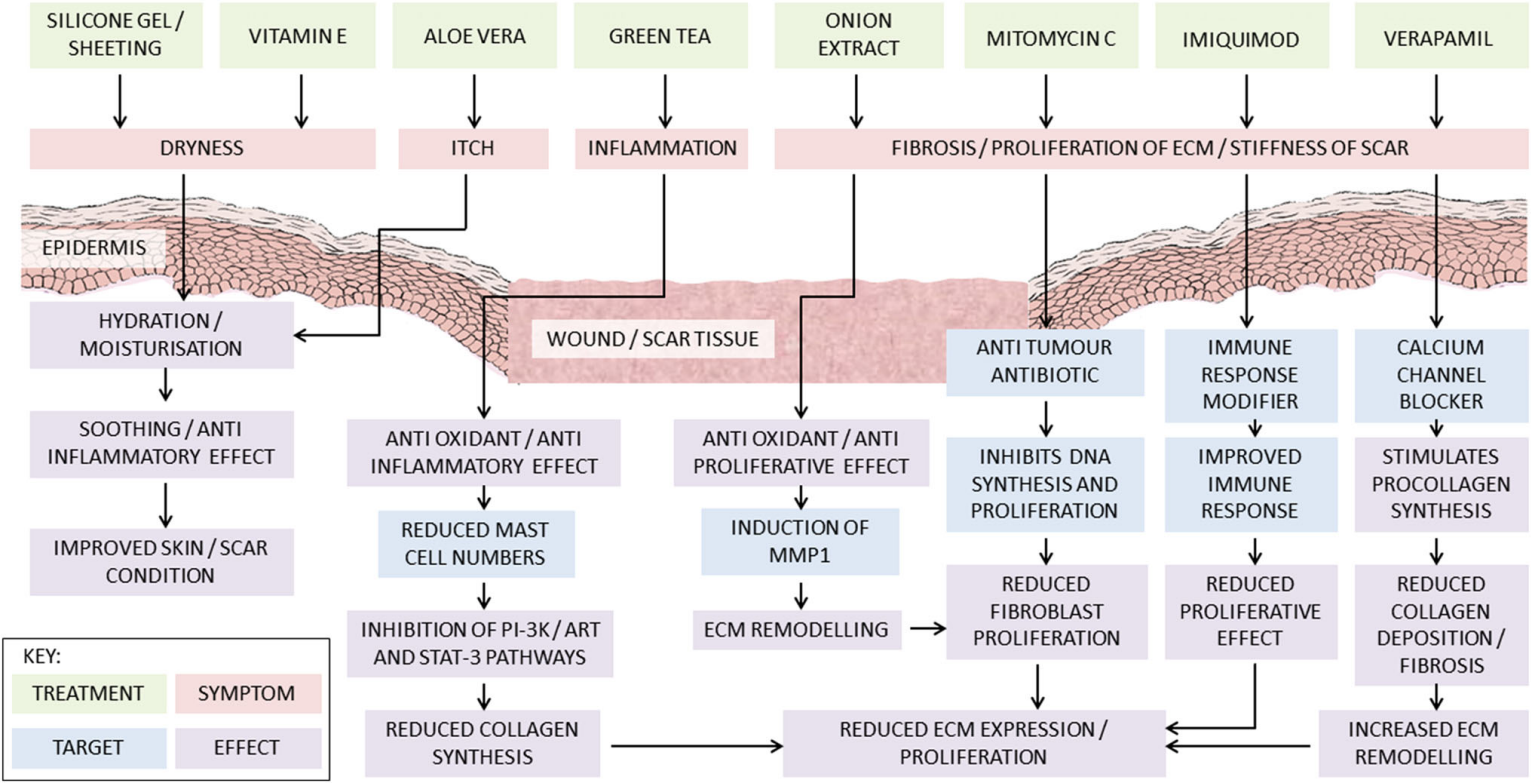
A flowchart depicting the effect of silicone gel and sheeting [1, 26, 92, 97, 117], vitamin E [36, 55, 66, 120], *Aloe vera* [39, 51, 67, 72], green tea [29, 40, 48, 71, 88, 109, 119, 121], onion extract [14, 24, 27, 31, 32, 34, 42, 43, 56, 59, 63, 65, 69, 79, 87, 91, 116], mitomycin C [6, 30, 53, 94, 96, 98, 103, 106, 110] and imiquimod [10–12, 23, 33, 73, 76, 89, 93, 105] at addressing symptoms commonly cited during wound healing, skin scarring and problematic abnormal raised dermal scarring such as keloid and hypertrophic scarring. The symptoms best targeted by the treatment are indicated, as well as the known mechanistic target and resulting effect in vivo

What is clear from this review is that a standardised and systematic approach and strategy for evaluating scars prior to deciding on the appropriate treatment regime is required (Fig. 2). Symptoms and signs, as well as physical and psychosocial complaints, need to be considered early, through use of objective scar assessment scales and possibly tools, to elucidate the most significant factors. The PRISM scale [18] potentially has additional benefit in this context, as it includes a patient’s perspective. Treatment, if appropriate, needs to address these specific issues individually (Table 3; Fig. 3). It is important to regularly monitor and re-evaluate response to therapy, particularly to assess signs and symptoms as they change in response to treatment and scar maturation. Thus, as symptoms and signs of the scars change over time, a clinician’s approach with targeted therapy would need to be altered. A greater utilisation of a range of subjective and objective non-invasive tools throughout this process, such as standardised photography, laser Doppler imaging, 3D cameras, and SIAscopy, would likely aid interpretation and evaluation of the skin scars, both in a research and clinical setting, and lead to a more targeted treatment based on managing problematic scar-related signs and symptoms.

**Table 3:** The known target and effect of available topical treatments in the processes of wound healing, skin scarring and abnormal raised dermal scarring such as keloid and hypertrophic scarring

Topical treatment	Effect
Silicone gel/sheeting [1, 26, 92, 97, 117]	Hydration and moisturisation
	Improved skin/scar condition
Imiquimod [10–12, 23, 33, 73, 76, 89, 93, 105]	Immune response modifier
	Reduced proliferative effect
	Reduced ECM expression
Mitomycin C [6, 30, 53, 94, 96, 98, 103, 106, 110]	Anti-tumour antibiotic
	Inhibits DNA synthesis and proliferation
	Reduced fibroblast proliferation
	Reduced ECM expression
Onion extract [14, 24, 27, 31, 32, 34, 42, 43, 56, 59, 63, 65, 69, 79, 87, 91, 116]	Anti-oxidant/anti-proliferative effect
	Induction of MMP1
	ECM remodelling
	Reduced fibroblast proliferation
Green tea [29, 40, 48, 71, 88, 109, 119, 121]	Anti-oxidant/anti-inflammatory effect
	Reduced mast cell numbers
	Inhibition of PI-3K/ART and STAT-3 pathways
	Reduced collagen synthesis
*Aloe vera* [39, 51, 67, 72]	Soothing/anti-inflammatory effect
	Hydration and moisturisation
	Improved skin/scar condition
Vitamin E [36, 55, 66, 120]	Hydration and moisturisation
	Improved skin/scar condition

Mechanisms of action abstracted from in vivo and in vitro data in currently available literature

## Abbreviations

ECM: Extracellular matrix
PDT: Photodynamic therapy
ES: Electrical stimulation
POSAS: Patient and Observer Scar Assessment Scale
TAC: Triamcinolone
5-FU: 5-Fluorouracil
EGCG: (−)-Epigallocatechin-3-gallate
MEBO: Moist exposure burn ointment
LOE: Levels of evidence
SIAscopy: Spectrophotometric intracutaneous analysis
